# Overview of detection methods of fetomaternal haemorrhage

**DOI:** 10.3389/fphys.2025.1445757

**Published:** 2025-04-11

**Authors:** Xinyang Li, Changfei Li, Tiemei Liu

**Affiliations:** ^1^ Department of Blood Transfusion, China-Japan Union Hospital of Jilin University, Changchun, Jilin, China; ^2^ Patient Service Center, The Second Hospital of Jilin University, Changchun, Jilin, China

**Keywords:** fetomaternal haemorrhage (FMH), detection method, hemolytic disease in newborns (HDN), fetal hemoglobin, flow cytometry

## Abstract

Fetomaternal haemorrhage is the same immunity that occurs when foetal and maternal blood are incompatible. It is critical to accurately quantify maternal haemorrhaging in order to prevent hemolytic disease in the infant. At this time, the rosette test and K-B test are the most frequently used techniques for detecting foetal red blood cells in the mother’s blood. However, the sensitivity of the rosette test is low, and due to its complex operation and high subjectivity, the K-B test cannot be used as a routine clinical detection method. This review therefore focuses primarily on the clinical landscape and future prospects of methods for detecting fetomaternal haemorrhage. In a general sense, this may bring to light the most promising strategy and encourage the development of technology for fetomaternal haemorrhage in order to guarantee the early detection and prevention of hemolytic disease in newborns.

## Introduction

Fetomaternal haemorrhage (FMH) is quantified primarily through the measurement of foetal red blood cells in the peripheral blood circulation of pregnant women (Simes et al., 2021; Piva et al., 2018; Biankin et al., 2003; Gică et al., 2021; Ficarola et al., 2022a; Simões et al., 2022; Piva et al., 2018). Addressing the issue of blood type incompatibility between the mother and foetus requires precise measurement of FMH. Preventing hemolytic disease in newborns (HDN) can be achieved through the administration of sufficient immunoglobulin (Hassan et al., 2019; Hubinont, 2016; Morris et al., 2016). It is determined by the quantity of foetal red blood cells transferred to the mother’s body whether or not to administer immunoglobulin. An excess of immunoglobulin will place a financial strain on the maternal family, while a deficiency will render prevention ineffective. Hence, a prenatal quantitative detection method that is effective, swift, uncomplicated, dependable, and precise is of utmost significance in the context of FMH.

Foetal red blood cells will continue to enter the mother’s body so long as FMH occurs due to a ruptured placenta, injury caused by haemorrhage, collision, abortion, or delivery (Ficarola et al., 2022b; Stroustrup and Trasande, 2012; Sinha et al., 2012; Meijer et al., 2023). Pregnant women will generate specific IgG antibodies against the surface antigen of foetal red blood cells if the blood group antigen on foetal red blood cells differs from that on pregnant women’s red blood cells (Piva et al., 2018). Such as anti-D, anti-Rh17 Antibody, anti-E and anti-Kell antibodies (Pal and Williams, 2015; Dajak et al., 2020). These antibodies can then cross-pollinate with foetal red blood cells via the placenta, resulting in the destruction of foetal red blood cells and subsequent hemolysis (Verduin et al., 2015). Since placental rupture first appeared at 16 weeks, there is a high risk of FMH for women who are pregnant for more than 16 weeks. David M determined via the K-B experiment that 22.5% of RhD-negative [RhD (−)] pregnant women experienced FMH during the delivery of a RhD-positive [RhD (+)] foetus (David et al., 2004). In cases where pregnant women who are RhD negative have RhD (+) foetal red blood cells, the RhD (+) foetal red blood cells induce the mother to generate IgG anti-D antibody (Ayenew, 2021; Bonstein et al., 2024). This antibody can then enter the foetus via the placenta, where it sensitises the foetal red blood cells, thereby inducing allogeneic immunity. Additionally, Kell antibody, anti-E antibody, and anti-C antibody are also responsible for HDN. FMH is calculated to be fatal for 3% of pregnant women, and a variety of complications may result from maternal haemorrhage, including anaemia, edoema, jaundice, bilirubin encephalopathy, and potentially fatal outcomes for the foetus or newborn (Tao et al., 2022; Liao et al., 2023; Sandilya et al., 2023; Stabile et al., 2023; Fortes et al., 2023a; Zheng et al., 2023). The primary rationales for implementing anti-D immunoglobulin treatment and early detection of FMH are to mitigate the risk of perinatal foetal morbidity and mortality (Khan et al., 2018; Dholakiya et al., 2021). Rh immunoglobulin (RhIg) (Slootweg et al., 2022; Flynn et al., 2022),which is in commercial supply, is a preparation of human immunoglobulin derived from the plasma of blood donors expressing high titers of anti-D antibodies (Tneh et al., 2021; Glazebrook et al., 2020; Slootweg et al., 2022; Biscoe and Kidson-Gerber, 2015; Günel et al., 2010; Serban et al., 2024; Raymond et al., 2023). This preparation has been shown to be effective in mitigating D-sensitization and subsequent hemolytic disease of the newborn (HDN) (Fortes et al., 2023b). Prior to the widespread implementation of FMH screening and anti-D immunoglobulin immunisation, newborn foetal mortality due to hemolytic disease in Britain and Wales stood at 1.2% in 1970. Following the implementation of FMH screening and anti-D immunoglobulin immunisation (Ramsey et al., 2023), the HDN-related foetal mortality rate decreased to 0.02%. Due to the fact that the appropriate dose is determined by the volume of FMH, addressing blood group incompatibility between the foetus and mother is crucial. Therefore, it is of the utmost importance for obstetrics and blood transfusion department administration to precisely quantify FMH prior to delivery and administer appropriate treatment.

In addition to providing a summary of the cases of FMH that have been discussed in earlier reviews, the objective of this review article is to provide an introduction to the clinical situation as well as the potential for new detection technology that is based on the results of prenatal quantitative preservation of FMH. In a general sense, this may bring to light the most promising detection strategy and encourage the development of maternal haemorrhage in order to prevent HDN.

### Rosette screen test

A highly sensitive method as a screening test to qualitatively determine the amount of FMH from 30 mL of peripheral maternal blood is the rosette screen test. Currently, this is the screening method approved by the FDA for clinical use. Sebring and Polesky initially documented in 1982 that foetal red blood cells could aggregate when incubated with enzyme-treated RhD indicator red blood cells and reagent anti-D serum (Sebring and Polesky, 1982). This observation was made possible under a microscope. This technique is restricted to identifying RhD (+) foetal cells and D-negative maternal cells (Titze et al., 2023). Peripheral red blood cells from pregnant women were combined with anti-D antibodies for an incubation reaction. Subsequently, any unbound anti-D antibodies that failed to engage in the reaction were removed by washing. Mix and resuspend O-type R_2_R_2_ red blood cell suspension before analysing the specimen under an optical microscope. When foetal RhD (+) red blood cells are present, the indicator cells will aggregate around the foetal cells to form rosettes. The experimental design was validated in the absence of agglutination and rosette in the negative control tube. The presence of a rosette ring and agglutination in the control tube signifies inadequate washing following incubation, which can lead to the agglutination of free anti-D red blood cells that are RhD positive indicator red blood cells. If the mother has weak D antigen, the rosette test may return a false positive result; conversely, if the foetus or newborn has weak D antigen, the result may return a false negative. Furthermore, in the event that the direct antiglobulin test (DAT) on the mother is positive, the antibody-coated cells on the mother will cross-link and agglutinate, potentially resulting in a false positive for the screening test.

300 mg of RhD immunoglobulin is sufficient to prevent immunisation in 99% of pregnant women with HDN if the rosette test is negative. A positive rosette test indicates that the volume of FMH surpasses 10 mL, and the level of FMH must be further quantified by the K-B test or flow cytometry before the RhD immunoglobulin dose required to prevent HDN can be administered (Dochez et al., 2024). When the foetal blood type is A, B, or AB and the pregnant woman has a naturally occurring isotype IgG antibody, foetal red blood cell destruction is more likely to occur (Fan et al., 2021). Hence, in order to anticipate blood group incompatibility between foetuses and pregnant women, it is imperative to conduct timely screening tests for FMH. As shown in Table 1, it is known that pregnant women can predict the foetal blood type.

**TABLE 1:** Relationship between maternal and fetal blood types.

Mother	Father	Children
A	A	A/O
	B	A/B/O/AB
	O	A/O
	AB	A/B/AB
B	A	A/B/O/AB
	B	B/O
	O	B/O
	AB	A/B/AB
O	A	A/O
	B	B/O
	O	O
	AB	A/B/AB
AB	A	A/B/AB
	B	A/B/AB
	O	A/B/AB
	AB	A/B/AB

The rosette screen test is inexpensive, straightforward, and uncomplicated to perform; it only requires a water bath, a centrifuge, and an optical microscope. As a result, the procedure can be executed at any moment, with a duration of 1–2 h. This experiment is not applicable to foetuses or newborns with a weak D phenotype (Pal and Williams, 2015). To detect FMH when determining the weak D phenotype of a foetus or newborn, the K-B test or flow cytometry with anti-fetal haemoglobin (anti-HbF) antibody should be utilised.

### The Kleihauer-Betke acid elution test

Kleihauer, Braun, and Betke initially delineated the Kleihauer-Betke acid elution test in 1957. The principle of the Kleihauer-Betke acid elution test (K-B test) for detecting FMH (Bonstein et al., 2024; Krywko et al., 2022), that is, the K-B test, is to distinguish foetal red blood cells from adult red blood cells according to the difference in acid resistance between fetal hemoglobin and adult hemoglobin (Stanic et al., 2020; Jia et al., 2023; Boller et al., 2021). Foetal red blood cells exhibit a pink hue subsequent to acid treatment, while adult red blood cells undergo a “ghost” appearance. Manual operation counting is the primary obstacle to the widespread application of the K-B experiment in clinical settings. Workers need to quickly distinguish foetal red blood cells from adult red blood cells according to their color, size and texture. Usually, the staff needs to count 2000 red blood cells within 20 min, and the K-B slide contains many substances such as neutrophils, overlapping cells and impurities, which increases the difficulty and subjectivity of the staff’s counting (Piva et al., 2018; Liggett et al., 2023). Therefore, high labor intensity, strong subjectivity and poor accuracy have become the shortcomings of K-B test (Audette et al., 2022; Zhang et al., 2021).

Based on the specific formula adopted, the correct dosage of RhIg can be determined by calculating the percentage of fetal cells identified in the K-B test to the equivalent volume of FMH. There are two specific methods as follows (Gereg and Fung, 2022):

(1) foetal hemorrhage volume (mL, hematocrit) = 2,400/(number of adult cells/number of foetal cells).
(2) First calculate the percent of fetal cell number: fetomaternal haemorrhage volume (mL, hematocrit) = foetal cell number (%) ×25.

Despite being the most commonly utilized FMH quantitative test in 2009, a significant majority of laboratories participating in CAP’s verification of foetal red blood cell detection capability employed the manual K-B test to quantify foetal red blood cells, or 95%). Furthermore, the K-B test is laborious and time-consuming (at least 2000 RBCs should be counted), and it is subjective. The results of analyzing the same blood sample by various individuals vary considerably. The potential for suboptimal accuracy and precision of the test exists so long as the pH value of the buffer employed and the thickness of the blood smear are considered. Additionally, the recommended RhIg dose and the variations in methods for calculating the volume of percent foetal cells as determined by the K-B test contribute to the variability. Table 2 of the AABB technical manual details the RhIg dosage for FMH.

**TABLE 2:** RhIG dosage of fetomaternal haemorrhage.

Fetal cell percentage (%)	Number of RhIg bottle	Dosage μg	IU
0.3∼0.8	2	600	3,000
0.9∼1.4	3	900	4,500
1.5∼2.0	4	1,200	6,000
2.1∼2.6	5	1,500	7,500

## Microcolumn gel technique

**FIGURE 1:**
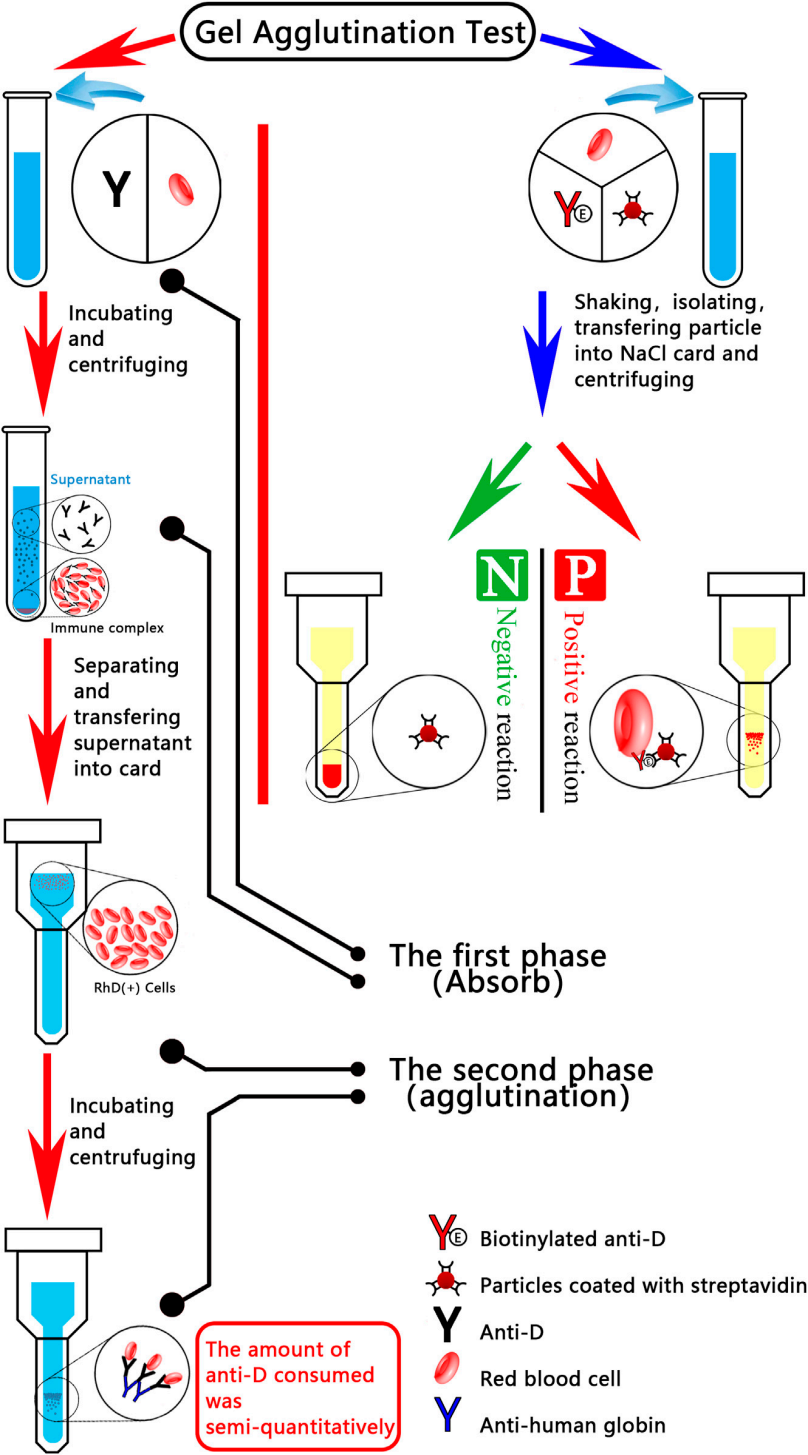
Diagrammatic representation of two microcolumn gel detection systems for fetomaternal haemorrhage. The red line indicates that, during the incubation phase, anti-D antibodies are applied to the washed maternal cells; RhD (+) foetal cells, if present, absorb the antibodies. Using an indirect anti-human globulin assay on standardised R2R2 cells, the residual anti-D in the supernatant was evaluated following incubation and centrifugation. Positive degree in the second stage provides a semi-quantitative indication of the quantity of anti-D consumed (i.e., binding to foetal cells in the first stage). A greater number of RhD (+) foetal cells (i.e., larger FMH) were present in the maternal sample, as indicated by the weaker reaction, which suggested that more anti-D was absorbed in the initial stage. An emerging particle gel immunoassay (PaGIA) for the quantification of FMH (FMH-PaGIA) is denoted by the blue line. ETDTA anticoagulant samples from D-negative pregnant women were combined with superparamagnetic particles that had been coated with monoclonal anti-D. Particles which have been separated using a magnetic particle concentrator are then placed in the gel card’s reaction chamber. The presence of D (+) cells, as indicated by agglutination particles or agglutination particles scattered across the gel matrix.

When washed maternal cells were subjected to incubation with anti-D antibodies, RhD (+) foetal cells gained antibodies. Anti-D residuals (in the supernatant) were evaluated using an indirect antiglobulin assay on standardised R_2_R_2_ cells following incubation and centrifugation. Anti-D antibody consumption is indicated semi-quantitatively by the positive degree in the second stage. A greater number of RhD (+) foetal cells are present in the maternal sample, as indicated by the weak reaction, which suggests that a greater number of anti-D antibodies are absorbed in the initial stage. New particle gel immunoassay methodology (Agaylan et al., 2007) was described by Agalan. EDTA anticoagulant samples from D-negative pregnant women were combined with superparamagnetic particles coated with monoclonal anti-D. Prior to placing the separated particles into the reaction chamber of the gel card, employ a magnetic particle concentrator. The presence of D (+) cells is indicated by agglutination particles or particles dispersed onto the gel matrix. Less than 0.1% is the sensitivity of the microcolumn gel method for detecting fetomaternal haemorrhage. According to Kimmittal, 77.5% of the fetomaternal haemorrhage from the patients was less than 4 mL, which corresponds to 0.2% of the fetomaternal haemorrhage (approximately 4 mL) that was identified using the microcolumn gel technique. In 22.5% of the patients, FMH measured 8.3 ± 1.7 mL, which corresponds to the 0.4% FMH (approximately 8 mL) that was identified using the microcolumn gel technique. Figure 1 illustrates the flowcharts of two microcolumn gel technologies designed for the purpose of FMH detection.

### Fluorescence microscope

Fluorescence microscopy are accurate and precise when it comes to quantifying FMH. 4 × 10^7^ red blood cells were resuspended in 200 μL of PBS subsequent to staining. The resuspended cells were subsequently examined utilizing a Zeiss fluorescence extension microscope. 256 cells were enumerated in 400× to ×100 times magnification in 256 small squares. In comparison to the K-B test, fluorescence staining offers enhanced levels of accuracy and precision. However, fluorescence microscopy is typically unavailable, costly, and require the expertise of trained personnel.

### Flow cytometry

Compared to the K-B test, the flow cytometer, which is capable of rapidly analysing 50,000 red blood cells, is equipped with an anti-foetal haemoglobin antibody (Weinhold et al., 2024; den et al., 2018; Kumpel et al., 2013; Quraishy and Sapatnekar, 2023; Peruzzi et al., 2024; Weinhold et al., 2024). The advantages of flow cytometry include high accuracy, specificity, and repeatability (Boller et al., 2021). Hence, flow cytometry possesses a greater breadth of clinical and research applications. Presently, one commercial kit has received FDA approval. This kit, manufactured by Fetaltrol, a division of Trillium Diagnostics based in Portland, ME, comprises a purified mouse monoclonal antibody targeting human HbF-IgG1 (Invitrogen, Camarillo, CA) and a distinct control kit for foetal red blood cells. Indodiphenyl nine dye (trichrome), fluorescein isothiocyanate (FITC), or R-phycoerythrin (R-PE) are bound by the antibody (Fibach, 2018). Consequently, the majority of published studies employed FITC-conjugated antibodies. The standard protocol entails the fixation of 2.5 × 3 × 107 red blood cells in 0.05% glutaraldehyde, the subsequent use of detergent (Triton X-100) to facilitate the penetration of antibodies through the cell membrane, and the subsequent combination with intracellular HbF. Davis et al. initially documented the permeabilization method utilizing glutaraldehyde and Triton X-100 for brief treatment, which enables the analysis of five to six samples within an hour (Davis et al., 1998). There is a propensity for cell aggregates to form in contrast to the permeation method of acetone and ethanol washing, which necessitates overnight incubation or numerous temperature-dependent steps. After staining the cells with antibodies, they were examined via flow cytometry. In order to differentiate the fluorescence of foetal red blood cells from the nonspecific background staining of autologous fluorescent white blood cells and cell fragments, it is advantageous to employ both positive and negative quality controls concurrently. In addition, positive control is useful for establishing the gating parameters of fetal cells. In 1999 and 2001, CAP surveys incorporated samples from both extremes of the threshold of 0.6% foetal cells or 30 mL foetal whole blood (15 mL fetal red blood cells). A 300 LG rhizome bottle contains 30 mL of foetal whole blood, which is considered adequate to prevent foetal red blood cells. The performance of laboratories employing anti-HbF flow cytometry or the K-B test is comparable for 0.4% of foetal cell samples; however, 50% of laboratories overestimate FMH, as evidenced by reported values greater than 0.6%. FMH was underestimated by over 10% of laboratories utilizing the K-B test for 0.8% of foetal cell samples, with a reported result of less than 0.6%. All laboratories employing anti-HbF flow cytometry accurately determined FMH. While the K-B test and anti-HbF flow cytometry exhibit a strong correlation in both small and large FMH, the published research aligns with the findings of the CAP capacity survey in that anti-HbF flow cytometry demonstrates superior experimental and inter-laboratory accuracy compared to the K-B test. The majority of research reports demonstrate that flow cytometry quantifies the volume of FMH with an accuracy of 0.1%. Conversely, the conventional flow cytometry approach necessitates the iterative washing of foetal red blood cells, resulting in their loss. The detection of FMH by APC-bound antibodies is described in Li’s article (Li et al., 2023). Flow cytometry was employed to detect EDTA anticoagulant in the peripheral blood of pregnant women. By utilizing hydrogel in conjunction with separation technology, this technology circumvents the need for multiple washing steps and addresses the issue of foetal red blood cell loss. This study presents the preliminary application of fluorescence immunoassay in conjunction with hydrogel medium for the diagnosis of FMH. The hydrogel fluoroimmunoassay identifies foetal haemoglobin in the peripheral blood of pregnant women by coupling anti-HbF antibodies with fluorescent antibodies and sensitised red blood cells to form immune complexes. The intensity of the fluorescence is measured using flow cytometry. The critical level of 0.1% can be detected by this technology. The anti-HbF antibody utilized in this study is a specific antibody targeting two distinct epitopes of HbF. The negative control sample, which is adult foetal hemoglobin, is utilized to measure the final fluorescence result of varying proportions of foetal hemoglobin. To mitigate the impact of Hb increase, the fluorescence value of FMH is subtracted from the fluorescence value of the negative control. Furthermore, the permeation step that is employed in the conventional anti-HbF flow cytometry method is excluded in this approach. In addition, false positive results are generated as a result of the inaccurate differentiation between maternal F cells and foetal haemoglobin (62). In healthy individuals, F cells contain a small amount of HbF, which ranges from 1% to 2%. These cells are regular red blood cells. Conversely, sickle cell anaemia and β-thalassemia, which are hereditary or acquired hemoglobinopathies, will result in a substantial increase in HbF content. In addition, F cells will be physiologically increased during pregnancy. Foetal red cells and adult red cells are distinguished by the distinct antigen specificity of haemoglobin in F cells and foetal red cells. As a result, the fluorescence intensities of these antigens are distinct due to their specificity. Nonetheless, flow cytometry is not currently utilized for screening purposes, primarily due to the fact that the majority of hospitals lack the equipment or the means to operate it continuously. Nevertheless, in medical facilities that possess adequate personnel and resources, anti-HbF flow cytometry appears to be a viable confirmation test. With any luck, future research will result in the development of a compact fluorescence analyzer capable of directly detecting the fluorescence intensity of the water-based rubber card bottom, as opposed to the current goal of reducing the size of the flow cytometer. The flow cytometer equipped with anti-fetal hemoglobin antibody, capable of rapidly analyzing 50,000 red blood cells, offers several advantages over the K-B test. These include enhanced precision, increased specificity, and improved repeatability. Hence, flow cytometry possesses a greater breadth of clinical and research applications.

FMH can also be quantified using anti-D antibody-based flow cytometry; however, its utility is restricted to clinical scenarios involving D antigen incompatibility. FDA-approved monoclonal anti-D antibodies are commercially available. Rh variants, specifically weak D and partial D, cannot be reliably detected by flow cytometry from D-negative cells; thus, false negative results may ensue. The variations of the Rh blood group system, which is extremely polymorphic, fall into two categories: weak D phenotype and partial D phenotype. Certain variants of D are distinguished by an extracellular domain mutation that results in an epitope change. On the contrary, the weak D mutation results from an intracellular or transmembrane domain mutation, which diminishes the quantity of D antigens exhibiting typical surface quality on red blood cells. While primary anti-D alloimmunization induced by weak D red blood cells is exceedingly improbable, an instance of alloimmunization during pregnancy via partial D antigen has been documented. These issues are circumvented by hydrogel fluoroimmunoassay technology.

### Gene identification

Pregnant women, especially those with poor or variant D, need to be managed according to their RhD genotype. If the result of RhD typing is a “serologic weak D phenotype”, the laboratory should retest the blood sample and refer it to a reference laboratory for RHD genotyping. If weak D type 1, 2 or 3 is not detected, the individual should be managed as RhD negative. If weak D type 1, 2 or 3 is detected, the individual can be safely managed as RhD positive. Until recently, it was thought that weak D individuals would not elicit an antibody response even if they encountered normal D antigen. However, it has been shown that some weak D phenotypes such as type 4 and type 15 can produce anti-D. Therefore, the view that variations other than weak D types 1, 2 and 3, which can be considered safe, should be accepted as partial D has gained weight. In conclusion, these pregnant women should actually be evaluated genotypically and those with weak D types 1,2,3 should be considered Rh positive and anti-D immunoglobulin treatment should not be administered.

### Application of an enzyme-linked immunosorbent assay utilizing a silk membrane

In this experimental setup, a silk membrane that has been coated with a particular antibody binds exclusively to the surface antigen of fetal red blood cells. This allows for the differentiation of fetal red blood cells from those of pregnant women, facilitating the quantification of fetal hemorrhage and the prevention of HDN. The permeability of the silk membrane was enhanced through a chemical treatment in this experiment. Subsequently, an immune complex comprising anti-fetal red blood cell antibody (anti-A antibody and anti-B antibody), cross-linked antibody, and anti-silk membrane antibody was utilized to react with the membrane and capture fetal red blood cells from the peripheral blood of pregnant women. After washing, biotin-avidin was introduced to enhance the visibility of the color development. The ELISA developed with silk membrane in the experiment is straightforward, rapid, and inexpensive; it is also non-hazardous to the health of pregnant and fetal organisms and requires no equipment or instruments. The outcomes are evident and straightforward to discern. An immunosensor analysis is the method by which antigens, antibodies, and analytes of interest are combined. It utilizes the three-dimensional and multi-layer pore structure of natural silk, in addition to its mechanical properties and immune activity. Red blood cells with an average diameter of 7.2 microns are able to traverse this structure without encountering any impediments. In this investigation, an electrospun fiber membrane could potentially substitute for the silk membrane. This alternative may offer enhanced sensitivity due to the nanofibers’ reduced diameter, increased surface area, and greater porosity. With the expectation that subsequent investigations will refine the detection process to enhance the method’s performance, and that suitable apparatus or devices will be developed to augment the test volume and facilitate the automated interpretation of findings, thereby obviating the need for manual operation and naked eye observation, this will guarantee the method’s formal validation and subsequent application in the clinic.

## Conclusion

Hydrogel fluoroimmunoassay technology, biosensors, and gene sequencing are novel technologies that are constantly emerging to FMH. However, due to certain limitations, a significant number of clinical experiments are required to verify them before they can be implemented in the clinic. The characteristics of FMH detection methods are compared in Table 3. At present, flow cytometry is a dependable detection technology. Nevertheless, certain municipal or county hospitals are unable to perform the procedure due to the instrument’s extremely high power consumption. A small instrument is anticipated to be created in order to detect fluorescent markers in subsequent experiments, rather than relying on flow cytometry.

**TABLE 3:** The classification and comparison of FMH testing methods.

Test method	Sensitivity	Characteristic	Cost
Rosette screen	high	qualitative	low
the K-B test	low	quantitative	low
Microcolumn gel technique	high	semiquantitative	high
Fluorescence microscope	high	qualitative	high
Flow cytometry	high	quantitative	high
Gene identification	high	---	high
An enzyme-linked immunosorbent assay utilizing a silk membrane	high	semiquantitative	low
